# Gonadal Transcriptome Analysis of Sex-Related Genes in the Protandrous Yellowfin Seabream (*Acanthopagrus latus*)

**DOI:** 10.3389/fgene.2020.00709

**Published:** 2020-07-16

**Authors:** Shizhu Li, Genmei Lin, Wenyu Fang, Peilin Huang, Dong Gao, Jing Huang, Jingui Xie, Jianguo Lu

**Affiliations:** ^1^School of Marine Sciences, Sun Yat-sen University, Zhuhai, China; ^2^Southern Marine Science and Engineering Guangdong Laboratory, Zhuhai, China

**Keywords:** yellowfin seabream, gonad transcriptome, sex reversal, sex-biased genes, protandrous hermaphroditic fish

## Abstract

Yellowfin seabream (*Acanthopagrus latus*), a protandrous hermaphroditic fish, is a good model for studying the mechanism of sex reversal. However, limited knowledge is known about the genetic information related to reproduction and sex differentiation in this species. Here, we performed *de novo* transcriptome sequencing analysis of the testis, ovotestis, and ovary to identify sex-related genes in yellowfin seabream. The results assembled 71,765 unigenes in which 16,126 and 17,560 unigenes were differentially expressed in the ovotestis and ovary compared to the testis, respectively. The most differentially expressed gene (DEG)-enriched Kyoto Encyclopedia of Genes and Genomes and GO pathways were closely associated with the synthesis of sex steroid hormones. Functional analyses identified 55 important sex-related DEGs, including 32 testis-biased DEGs (*dmrt1*, *amh*, and *sox9*, etc.), 20 ovary-biased DEGs (*cyp19a*, *foxl2*, and *wnt4*, etc.), and 3 ovotestis-biased DEGs (*lhb*, *dmrt2*, and *foxh1*). Furthermore, the testis-specific expression of *dmrt1* and the brain-pituitary-ovary axis expression of *foxl2* were characterized, suggesting that they might play important roles in sex differentiation in yellowfin seabream. Our present work provided an important molecular basis for elucidating the mechanisms underlying sexual transition and reproductional regulation in yellowfin seabream.

## Introduction

Sex is one of the most intriguing propositions in life science (Mei and Gui, 2015; Schubert, 2015; Li and Gui, 2018). Usually, once the sex is determined, it will be maintained forever in animals. However, sex reversal has been found in a few lower animals, especial in fish species (Todd et al., 2016). Fish are the most species-rich group of vertebrates that have diverse sex strategies. From the discovery of hermaphrodite and sex reversal in rice field eel (*Monopterus albus*; Liu, 1944), numerous significant achievements have been obtained in basic studies on fish sex (Mei and Gui, 2015). It has been reported that there are about 500 hermaphrodite species (Avise and Mank, 2009). The hermaphrodites could be classified to three types: protogynous in grouper (*Epinephelus coioides*) and wrasse (*Thalassoma bifasciatum*), protandrous in Asian seabass (*Lates calcarifer*), and black seabream (*Acanthopagrus schlegelii*), and bidirectional hermaphrodites in *Gobiodon erythrospilus*. Fish have the most diverse sex-determining strategies, including environmental sex determination (ESD), and genotypic sex determination (GSD), such as XX/XY, and ZZ/ZW systems and their variants as well sex-related extra chromosomes (Li et al., 2016, 2017; Li and Gui, 2018).

The SD genes seem to be conserved with *Sry* for mammals and *Dmrt1* for bird (Koopman et al., 1991). However, the SD genes are highly variable in fish species. Recently, advances in fish SD have been fueled by the rapid development of affordable genome and transcriptome technologies (Kamiya et al., 2012; Myosho et al., 2012; Yano et al., 2012; Chen et al., 2014; Dan et al., 2018). *Dmy* (doublesex- and mab-3-related transcription factor 1-Y) is the first identified SD gene in fish (Matsuda et al., 2002). Subsequently, different SD genes are revealed in other fish species, such as *amhy* (Y-linked anti-Mullerian hormone) in *Odontesthes hatcheri* (Hattori et al., 2012), *amhr2* (anti-Mullerian hormone receptor type 2) in *Takifugu rubripes* (Kamiya et al., 2012), *sdY* (sexually dimorphic on the Y chromosome) in *Oncorhynchus mykiss* (Yano et al., 2012), and *pfpdz1* in *Pelteobagrus fulvidraco* (Dan et al., 2018). Interestingly, the SD genes might be different even in the same fish subspecies, such as *dmy* in *Oryzias latipes* (*O. latipes*; Matsuda et al., 2002), *gsdf* (gonadal somatic cell derived factor) in *O. luzonensis* (Myosho et al., 2012), and *sox3-Y* (SRY-box transcription factor 3-Y) in *O. dancena* (Takehana et al., 2014). To date, SD and sexual differentiation are mainly investigated in gonochoristic species; nevertheless, limited knowledge is known in hermaphroditic fish. Recently, several known SD-related genes have been cloned in several hermaphroditic fish, such as *foxl2*, and *gsdf* in *M. albus* (Hu et al., 2014; Zhu et al., 2016), *dmrt1* and *sox3* in *E. coioides* (Xia et al., 2007; Yao et al., 2007), and *amh* and *amhr2* in *A. schlegelii* (Wu et al., 2010). Whereas the sex-related signaling pathways remain unclear, and their conservation should be analyzed in other hermaphroditic animals.

Sparidae is a group of teleost fish with different reproductive strategies, including gonochorism and hermaphroditism. For example, the common dentex (*Dentex dentex*) is a gonochoristic species (Tsakogiannis et al., 2018a), red porgy (*Pagrus pagrus*), and common pandora (*pagellus erythrinus*) are protogynous hermaphrodites (Tsakogiannis et al., 2018b), gilthead seabream (*Sparus aurata*), and yellowfin seabream (*Acanthopagrus latus*) are protandrous hermaphrodites (Negintaji et al., 2018; Tsakogiannis et al., 2018a), and sharpsnout seabream (*Diplodus puntazzo*) is a rudimentary hermaphrodite fish species (Papadaki et al., 2018). Therefore, Sparidae is a good model for studying SD and sex reversal.

Yellowfin seabream (*A. latus*) belonging to the Sparidae family is a commercially important fish that is popular in Asia because of its beautiful appearance and delicious taste. Yellowfin seabream initially develops a functional testis during the first reproductive cycle; then it undergoes a long term of sexual reversal to develop a functional ovary in the third year. Therefore, yellowfin seabream is considered as a good model for studying sex differentiation and sex reversal. However, there is little information about gonadal development and sex-related genes in this species. In this study, aiming to explore the genetic information associated with gonadal differentiation and sex reversal in yellowfin seabream, we carried out transcriptome sequencing analysis of testis, ovotestis, and ovary. Our results identified a large number of sex-related candidates and the dynamics of signaling pathway during sex reversal, which would provide a molecular basis for studying the mechanism underlying sexual transition and reproduction regulation in this species.

## Materials and Methods

### Ethical Procedures

All experimental procedures in our study with *A. latus* were approved by the Ethics Committee of Sun Yat-sen University.

### Sample Collection and Histological Examination

Yellowfin seabreams were provided from Hailv aquaculture station (Zhuhai, China). Nine of the yellowfin seabreams were chosen and divided into 3 samples in triplicates for the subsequent experiments, including three 1-year-olds with average body weight (ABW) of 113.3 ± 8.5 *g* and average body length (ABL) of 15.3 ± 0.3 cm, three 2-year-olds (ABW = 226.7 ± 21.6 *g*, ABL = 19.5 ± 0.6 cm), and three 3-year-olds (ABW = 519.3 ± 55.3 *g*, ABL = 26.1 ± 0.8 cm). Each fish was anesthetized by MS-222 (Sigma, United States), and half of the gonads were fixed in 4% PFA/PBS for later histological analysis, and the other was frozen with liquid nitrogen for RNA extraction. Subsequently, the fixed gonad were embedded in paraffin, sectioned, and stained with hematoxylin and eosin (HE). The images were acquired with an inverted microscope (Zeiss, Germany).

### RNA Extraction and Illumina Sequencing

Total RNA was isolated with a combined method of trizol (Invitrogen, United States) and SV (Spin or Vacuum) total RNA isolation system (Promega, Unites States), which could yield high-quality RNA. The samples were homogenized with 1 ml trizol reagent for 40 s with BioPrep-24 (Tomos, China), followed by adding 200 μl chloroform and mixed thoroughly. After incubating at room temperature for 5 min, samples were centrifuged at 10,000 × *g* for 15 min at 4°C; then the upper phase (about 400 μl) was transferred to a new nuclease-free tube and mixed thoroughly with 200 μl ethanol. The mixture was transferred to a new spin column and centrifuged at 14,000 × *g* for 1 min at room temperature. The following steps were performed according to the manufacturer’s instructions of the SV total RNA isolation system. The amount and quality of isolated RNA were determined by BioSpec-nano Spectrophotometer (Shimadzu, Japan) and Agilent 2100 Bioanalyzer (Agilent Technologies, United States). High-quality RNA (28S/18S ≥ 1.5, OD260/280 ≥ 1.8, and RIN value ≥ 7) was used for cDNA library construction. The library was generated as previously described (Lin et al., 2020). The *de novo* transcriptome sequencing was performed on the Illumina Hiseq platform (Shanghai Majorbio Bio-pharm Technology Co., Ltd., China). The data have been submitted to the Genbank (SRA accession number: PRJNA622226).

### Transcriptome Assembly and Gene Annotation

After sequencing, raw reads were filtered with three steps to get the high-quality clean reads. First, SeqPrep software^1^ was used to remove adapter sequences. The sickle software^2^ was used to trim and discard the low-quality reads with a Q-score less than 10. Next, raw reads with poly-N (N means ambiguous base) or length less than 30 bp were discarded. The clean data of the nine samples were pooled together for *de novo* assembly with Trinity^3^ with optimization by TransRate^4^, CD-HIT^5^, and BUSCO^6^. The assembled sequences were annotated to public databases of NCBI_Nr, Swiss-Prot, and clusters of orthologous groups of proteins (COG) with a threshold *E*-value < 10^–5^. Further functional annotation were performed against the Pfam database with HMMER3, gene ontology (GO) with BLAST2GO (version 2.5.0), and Kyoto Encyclopedia of Genes and Genomes (KEGG) with KOBAS (version 2.1.1), respectively.

### Differential Expression Analysis

The gene expression levels were standardized by fragments per kilobases per million (FPKM) read values with RSEM software^7^. The expressed genes were filtered with a threshold of FPKM value greater than 0.5. The analysis of differentially expressed genes (DEGs) was performed by DESeq2 software with BH method (false discovery rate correction with Benjamini/Hochberg) with “adjusted *p*-value < 0.05 and the absolute value of log2 fold change ≥ 1” as a threshold to judge the significance of gene expression difference. The DEGs in sex-related GO terms and KEGG pathways were considered as sex-related genes.

### Reverse Transcribed and Real-Time Quantitative PCR (RT-qPCR) Verification

To validate the transcriptome data, first-strand cDNAs were synthesized, and Real-Time Quantitative PCR (RT-qPCR) analyses were performed with the RNA template used for transcriptome sequencing. Total RNA from triplicated individuals in each age group were mixed in equal mass for reverse transcription. The first-strand cDNAs were transcribed with the PrimeScript^TM^ RT reagent Kit (Takara, Japan) according to the manufacturer’s instruction. Primers were designed by Primer3^8^. RT-qPCR was performed as previously described (Lin et al., 2020). Briefly, the thermal cycling conditions were: 94°C for 2 min, 40 cycles of 94°C for 15 s, 57°C for 15 s, 72°C for 15 s, and an additional 72°C for 2 min, followed by a final disassociation curve analysis. All the samples were analyzed in triplicates, and relative expression levels were normalized to β*-actin* by using the 2^–ΔΔCT^ methods. Primer information is listed in Supplementary Table S1.

### Statistics Analysis

All statistics were calculated using SPSS version 20. Differences between control and treatment groups were assessed by one-way ANOVA. *p* < 0.05 was considered as significant difference.

## Results

### Morphological and Histological Structure of the Testis, Ovotestis, and Ovary

**FIGURE 1:**
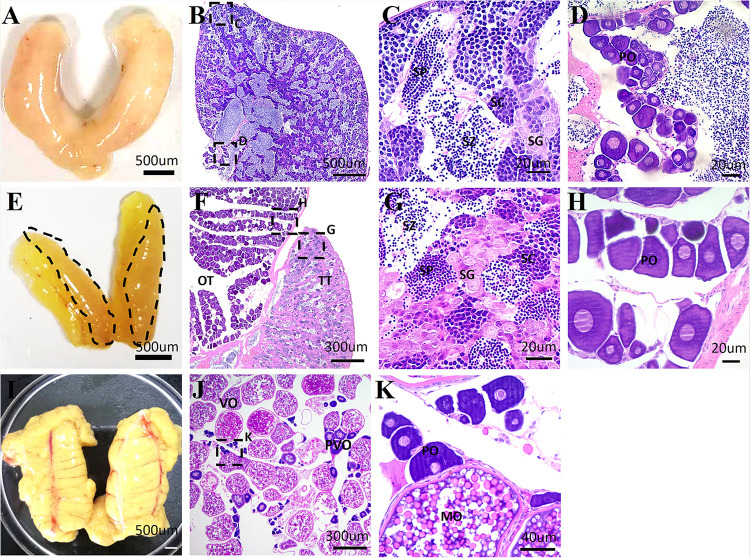
The representative images of the morphology and histological structures of the testis, ovotestis, and ovary of yellowfin seabream. The morphology of testis **(A)**, ovotestis **(E)**, and ovary **(I)**. The dotted line in panel **(E)** indicates the testicular portion of ovotestis. HE staining of the testis **(B–D)**, ovotestis **(F–H)**, and ovary **(J,K)**. **(C,D)** The magnified images in panel **(B)**; **(G,H)** the magnified images in panel **(F)**; and **(K)** the magnified images in panel **(J)**. MO, mature oocyte; OT, ovarian tissue; PO, primary oocyte; PVO, previtellogenic oocyte; SC, spermatocyte; SG, spermatogonia; SP, spermatid; SZ, spermatozoa; TT, testicular tissue; VO, vitellogenic oocyte.

Although the occurrence of sex reversal in Sparidae is known, the gonadal structure remains unclear in yellowfin seabream. To clarify it, HE staining was performed to characterize the structure of testis, ovotestis, and ovary in 1-, 2-, and 3-year-old yellowfin seabream, respectively. In 1-year-old yellowfin seabream, the milky and swollen gonads were comprised of a huge testicular portion containing all stages of male germ cells and numerous sperm as well as a very small ovarian portion with some primary oocytes (Figures 1A–D). After the first breeding season, the mature testis gradually degenerated; meanwhile, the ovary started to grow and develop. In two-year-old yellowfin seabream, the ovotestis was divided into two distinct portions: a distinct pale orange red and translucent testis comprising a loose structure with many spermatogonias and a faint yellow ovary consisting of many primary oocytes and previtellogenic oocytes (Figures 1E–H). In 3-year-old fish, the mature ovary is filled with lots of mature oocytes, and vitellogenic oocytes as well as a few primary oocytes and previtellogenic oocytes (Figures 1I–K). Next, we performed *de novo* transcriptome sequencing analysis of the testis, ovotestis, and ovary.

### Transcriptome Sequencing, Assembly, and Annotation

**FIGURE 2:**
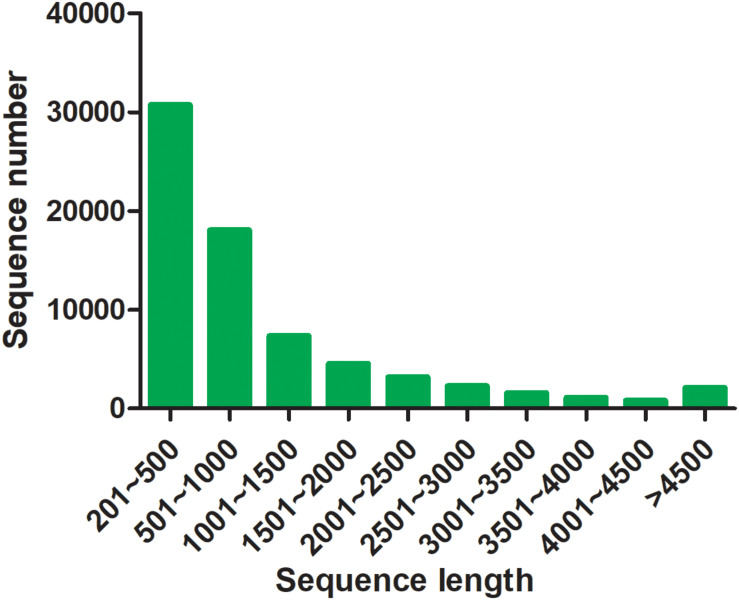
Length distribution of assembled unigenes.

By transcriptome sequencing, 10.1, 7.9, and 8.5 Gb raw data were obtained from the testis, ovotesits, and ovary, respectively. After filtering low-quality reads, 9.8, 7.7, and 8.3 Gb of clean data were obtained in testis, ovotestis, and ovary with Q20 values of 98.68, 98, and 98.25%, respectively (Supplementary Table S2). All of the transcriptomes were grouped into a single file for redundancy elimination. As a result, 71,765 unigenes were assembled, which had an average length of 1201 bp, and an N50 length of 2032 bp (Supplementary Table S3). Length distribution analysis showed that 48,763 unigenes (68%) ranged from 200 to 1000 bp; 17,354 unigenes (24%) ranged from 1000 to 3000 bp, and the remained 5648 unigenes (8%) were longer than 3000 bp in length (Figure 2). The unigenes were functionally annotated to the multiple public databases by BLAST with a threshold *e* value less than 10^–5^. A total of 30,584 (42.62%) unigenes were matched with the public databases: in which 29,252 (40.76%), 25,425 (35.43%), 22,732 (31.68%), 13,183 (18.37%), 9522 (13.27%), and 20,057 (27.95%) were matched in NCBI_Nr, Swiss-Prot, Pfam, COG, GO, and KEGG databases, respectively, (Supplementary Table S4).

### Quantification and Differentially Expressed Analysis of Unigenes

**FIGURE 3:**
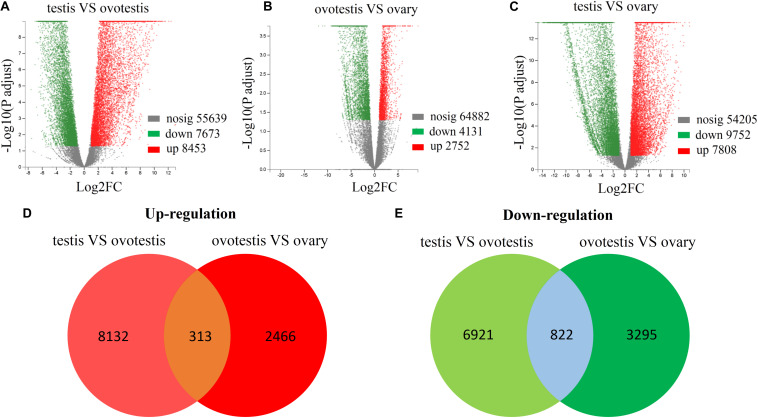
Differential expression analysis of unigenes. Volcano plot of differentially expressed unigenes between testis and ovotestis **(A)**, ovotestis and ovary **(B)**, testis and ovary **(C)**. The up-regulated, down-regulated DEGs were respectively shown in red and green dots, whereas not differentially expressed genes were indicated in gray dots. The venn diagrams of up-regulated **(D)** or down-regulated **(E)** DEGs between testis vs ovotestis and ovotestis vs ovary.

Subsequently, comparative analyses were performed to obtain evidence-based insights into sex reversal. A total of 22,591 DEGs were obtained in the testis, ovotestis, and ovary. Compared to the testis, there were 8453 upregulated DEGs and 7673 downregulated DEGs in the ovotestis and 7808 upregulated DEGs and 9752 downregulated DEGs in the ovary. Compared to the ovotestis, there were 2752 upregulated DEGs and 4131 downregulated DEGs in the ovary (Figures 3A–C). Moreover, there were 313 continuously upregulated DEGs and 822 downregulated DEGs during sex reversal process from testis over ovotestis to ovary (Figures 3D,E). Furthermore, the top 10 up-, or downregulated genes were screened between the ovotestis and testis (Table 1). Several of them were reported to be associated with spermatogenesis. For example, *kelch-like 10* (*klhl10*) is required for the development of elongated spermatids, and epididymal spermatozoa (Yan et al., 2004). *Tektin2* is expressed in spermatozoa and might be associated with sperm motility (Xiong et al., 2018). It has been reported that radial spoke head protein (RSPH) family contribute to sperm motility in human (Jeanson et al., 2015). Therefore, *rsph10b* might also be associated with sperm development in yellowfin seabream. In addition, it has been reported that mutation of *cfap251* leads to immotile sperm and infertility in men (Auguste et al., 2018). Our present results reveal that *klhl10*, *rsph10b*, *tektin2*, and *cfap251* were highly expressed in testis. In addition, the top 10 up- or downregulated genes between the ovotestis and testis, the ovary and ovotestis are, respectively, listed in Supplementary Tables S5, S6.

**TABLE 1:** Top 10 up- or downregulated genes in the ovotestis compared with the testis.

Sequence ID	Annotation	Gene name	Log_2_FC (ovotestis/testis)
DN35544_c0_g1	Fatty acid-binding protein, adipocyte-like	*Fabp*	11.56
DN23463_c0_g1	Probable pancreatic secretory proteinase inhibitor isoform X2	*Pspi*	10.64
DN35542_c0_g1	Toll-like receptor 2 type 2	*Tlr2*	10.03
DN41698_c3_g2	Receptor-type tyrosine-protein phosphatase zeta	*Ptprz*	9.65
DN77551_c0_g1	Krueppel-like factor 7	*Klf7*	9.41
DN27243_c0_g1	Synaptic vesicle 2-related protein-like	*Svop2l*	9.07
DN19358_c0_g1	Transposase	—	9.07
DN33418_c0_g1	Cytochrome c oxidase subunit 8A	*Cco8a*	9.03
DN25326_c0_g1	Homeodomain-interacting protein kinase 1-like	*Hipk1l*	9
DN32989_c0_g1	Pyroglutamyl-peptidase 1-like	*Pgpep1l*	8.99
DN27187_c0_g1	Cytochrome c oxidase subunit 3	*Cco3*	−7.59
DN29618_c0_g1	NADH dehydrogenase subunit 4	*Nd4*	−7.51
DN41537_c1_g2	Ras-related protein Rab-28	*Rab28*	−7.02
DN42167_c0_g1	Radial spoke head 10 homolog B2 isoform X2	*Rsph10b*	−7.02
DN38767_c0_g2	Nucleolar protein 4-like	*Nol4l*	−6.85
DN56599_c0_g1	Cytochrome c oxidase subunit 1	*Cco1*	−6.8
DN31297_c0_g1	Kelch-like protein 10	*Klhl10*	−6.8
DN36501_c0_g1	Zinc fingers and homeoboxes protein 1	*Zhx1*	−6.75
DN43960_c2_g2	Tektin-2	*Tekt2*	−6.65
DN37919_c0_g1	Cilia- and flagella- associated protein 251	*Cfap251*	−6.61

*The genes above and below the line are upregulated and downregulated in the ovotestis compared with the testis, respectively.*

### Analysis of the DEGs Associated With GO and KEGG Pathways of Sexual Differentiation and Gonadal Development

**FIGURE 4:**
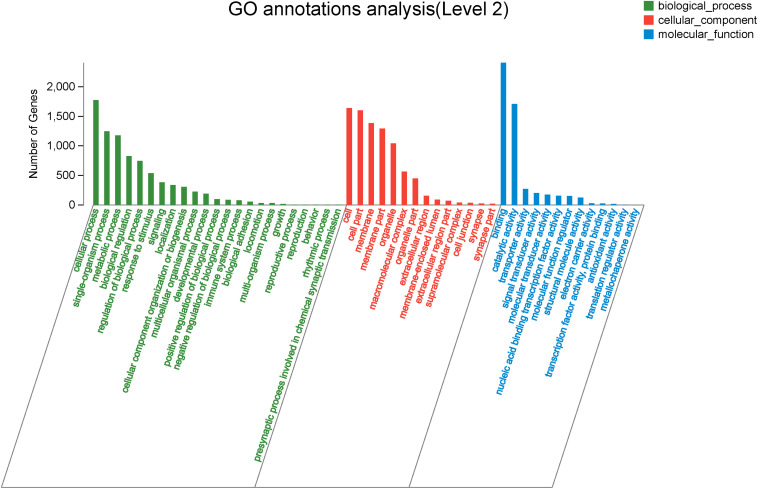
The Gene Ontology annotation of all DEGs. The X-axis represents the 50 subcategories. The Y-axis indicates the number of DEGs in a specific function cluster.

**FIGURE 5:**
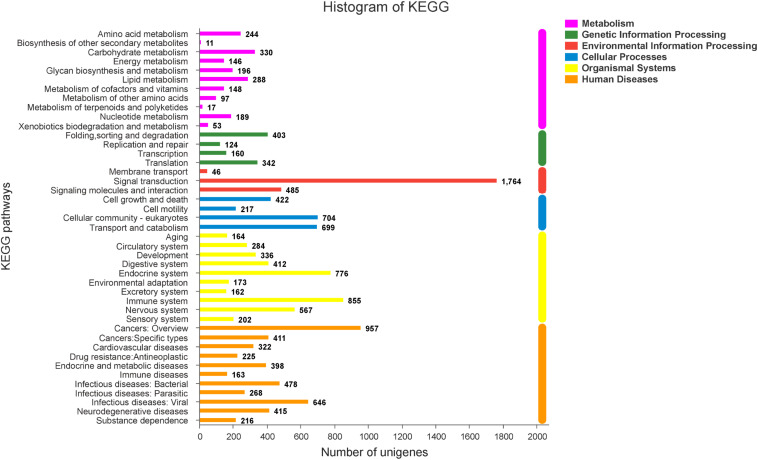
The KEGG classification of DEGs.

To explore genes associated with sexual differentiation and gonadal development, sex-related GO annotation, and KEGG pathway analysis were searched for all DEGs. The results showed that 4563 and 5797 DEGs were matched to 50 GO and 318 KEGG subcategories, respectively, (Figures 4, 5). Many DEGs were found to be involved with sex-related GO terms, such as gamete generation, fertilization, sex differentiation, and meiotic cell cycle (Table 2). The expression of spermatogenesis-related DEGs were significantly decreased in the ovotestis and ovary, such as deleted in azoospermia-like (*dazl*), sperm surface protein (*sp17*), cytochrome P450 family 17 subfamily a (*cyp17a*), anti-mullerian hormone (*amh*), and testis-expressed sequence 11 protein (*tex11*), whereas the expression of oogenesis-related genes, including spindling-1 like (*spin1l*), and bone morphogenetic protein 15 (*bmp15*) were significantly upregulated during sex reversal. The most DEG enriched sex-related KEGG pathways were associated with steroid production, including the steroid hormone biosynthesis (ko00140), steroid biosynthesis (ko00100), and ovarian steroidogenesis (ko04913) pathways (Table 3). It is known that sex determination and maintenance are dependent steroid hormones (Senthilkumaran, 2015; Gennotte et al., 2017), the production of which is regulated by cytochrome P450 (cyp), steroid sulfatase, and hydroxysteroid dehydrogenases (hsd; Uno et al., 2012; Hilborn et al., 2017). The expression of seven cyp members (*cyp7b*, *cyp21a*, *cyp11b1*, *cyp11a*, *cyp17a*, *cyp51*, and *cyp27b*) and *hsd17b2* were significantly downregulated during sex reversal, whereas the expression of *cyp3a*, *cyp2j*, *cyp19a*, *hsd17b1*, and *hsd17b12* were significantly upregulated. The wnt signaling pathway is required for early ovary development and folliculogenesis in mammal (Hernandez Gifford, 2015). The results indicate that the expression of wnt signaling components, such as *wnt4*, *lef1*, *wnt9b*, *wnt16*, *wnt5a*, and *axin2*, were significantly increased during testis-ovotestis-ovary sex reversal. In addition, two members of TGF-β signaling, *amh*, and its receptor *amhr*, were preferentially expressed in the testis.

**TABLE 2:** The representative DEGs involved in sex-related GO terms.

GO term (GO ID)	Sequence ID	Annotation	Gene name	FPKM			Sex-bias
				
				Testis	Ovotestis	Ovary	
Gamete generation (GO: 0007276)	DN43077_c1_g2	Progesterone receptor	*pgr*	1.26	0.09	0.05	Testis
	DN26672_c0_g1	Membrane progestin receptor alpha	*mpgra*	8.18	0.08	0.14	Testis
	DN34223_c1_g1	Deleted in azoospermia like	*dazl*	321.89	50.33	70.73	Testis
	DN42630_c0_g1	Spindlin-1-like	*spin1l*	0.88	1.05	2.57	Ovary
Fertilization (GO: 0009566)	DN40812_c0_g1	Sperm surface protein 17	*sp17*	4.08	0.21	0.64	Testis
	DN29158_c1_g1	Sperm acrosome membrane associated protein 6	—	60.74	0.4	0	Testis
Sex differentiation (GO: 0007548)	DN32655_c1_g1	Cytochrome P450 family 17 subfamily a	*cyp17a*	8.27	2.32	4.08	Testis
	DN31317_c0_g1	Anti-mullerian hormone	*amh*	2.08	0.52	0.07	Testis
	DN2559_c0_g1	Bone morphogenetic protein 15	*bmp15*	21.35	108.22	251.75	Ovary
Meiotic cell cycle (GO: 0051321)	DN41779_c0_g4	Testis-expressed sequence 11 protein	*tex11*	3.78	0.06	0	Testis

**TABLE 3:** The representative DEGs involved in sex-related KEGG pathway.

KEGG pathway (ID)	Sequence ID	Annotation	Gene name	FPKM			Sex-bias
				
				Testis	Ovotestis	Ovary	
Steroid hormone biosynthesis (ko00140)	DN64550_c0_g1	Cytochrome P450 family 7 subfamily b	*cyp7b*	1.12	0.09	0.04	Testis
	DN40933_c0_g1	Cytochrome P450 family 21 subfamily a	*cyp21a*	63.69	3.73	0.46	Testis
	DN32494_c0_g1	Cytochrome P450 family 11 subfamily b	*cyp11b1*	6.66	0.81	0.01	Testis
	DN30920_c0_g1	Cytochrome P450 family 11 subfamily a	*cyp11a*	1.88	0.19	0.18	Testis
	DN32655_c1_g1	Cytochrome P450 family 17 subfamily a	*cyp17a*	8.27	2.32	4.08	Testis
	DN43041_c0_g2	17β-hydroxysteroid dehydrogenase type 2	*hsd17b2*	12.57	0.86	2.95	Testis
	DN29241_c0_g1	Aldo-keto reductase family 1 member d1	*akr1d1*	8.09	0.53	0.07	Testis
	DN29283_c0_g1	Cytochrome P450 family 17 subfamily a	*cyp3a*	0.98	0.52	2.57	Ovary
	DN35942_c0_g2	Cytochrome P450 family 19 subfamily a	*cyp19a*	0.18	0.34	0.45	Ovary
	DN36375_c0_g1	17β-hydroxysteroid dehydrogenase type 1	*hsd17b1*	0.36	4.05	21.92	Ovary
	DN27313_c0_g1	17β-hydroxysteroid dehydrogenase type 12	*hsd17b12*	2.22	14.71	22.32	Ovary
Steroid biosynthesis (ko00100)	DN42791_c0_g1	Cytochrome P450 family 51	*cyp51*	11.23	0.65	1.88	Testis
	DN32572_c0_g1	Cytochrome P450 family 27 subfamily b	*cyp27b*	0.84	0.21	0.09	Testis
Ovarian steroidogenesis (ko04913)	DN30488_c0_g1	Steroidogenic acute regulatory protein	*star*	8.45	0.85	0.75	Testis
	DN43734_c1_g2	Follicle stimulating hormone receptor	*fshr*	46.23	2.81	1.16	Testis
	DN22210_c0_g1	Luteinizing hormone beta	*lhb*	1.56	48.37	38.08	Ovotestis
	DN43713_c4_g1	Cytochrome P450 family 2 subfamily j	*cyp2j*	32.18	144.62	241.48	Ovary
	DN25129_c0_g1	Growth differentiation factor 9	*gdf9*	2.33	50.99	103.6	Ovary
TGF-beta signaling pathway (ko04350)	DN40695_c0_g1	Anti-mullerian hormone type 2 receptor	*amhr2*	9.36	2.04	2.43	Testis
	DN31317_c0_g1	Anti-mullerian hormone	*amh*	2.08	0.52	0.07	Testis
Wnt signaling pathway (ko04310)	DN56662_c0_g1	Wingless-type MMTV integration site family member 4	*wnt4*	0	0.39	0.66	Ovary
	DN25748_c0_g1	Lymphoid enhancer-binding factor 1	*lef1*	1.3	2.23	5	Ovary
	DN21056_c0_g1	Wingless-type MMTV integration site family member 9b	*wnt9b*	0.14	0.18	0.78	Ovary
	DN42445_c1_g1	Wingless-type MMTV integration site family member 16	*wnt16*	2.04	3.92	9.57	Ovary
	DN44065_c1_g1	Wingless-type MMTV integration site family member 5a	*wnt5a*	0.25	0.52	4.11	Ovary
	DN32550_c0_g1	Axis inhibition protein 2	*axin2*	1.11	0.32	3.98	Ovary

### Analysis of DEGs Associated With Sex Determination and Sex Differentiation

Furthermore, genes associated with SD and gametogenesis were comparatively analyzed (Table 4). The expression of testis-determining genes were significantly decreased during sex reversal, such as SRY-box containing protein 9a (*sox9a*), *sox9b*, *sox10*, doublesex, and mab-3 related transcription factor 1 (*dmrt1*), *dmrt3*, and *dmrt4*. Meanwhile, the spermatogenesis-associated markers, piwi-like RNA-mediated gene silencing 1, 2 (*piwil1*, *2*); synaptonemal complex protein 1, 2, 3, (*sycp1-3*); and outer dense fiber protein 2, 3 (*odf2*, *3*), were also downregulated during the sex reversal. Whereas the expression of known ovarian differentiation-related genes were increased during sex reversal, for example, forkhead transcription factor L2 (*foxl2*), *sox3*, and *dax1*. Two oocyte markers, factor in the germline alpha (*figla*) and zona pellucida glycoprotein 3 (*zp3*), were dramatically upregulated in ovotestis and ovary. Overall, we identified 55 sex-related candidate genes, including 32 testis-biased genes, 20 ovary-biased genes, and 3 ovotestis-biased genes through these three strategies.

**TABLE 4:** Screening of sex determination and sex differentiation associated genes in yellowfin seabream.

Sequence ID	Annotation	Gene name	FPKM			Sex-bias
			
			Testis	Ovotestis	Ovary	
DN40316_c0_g1	SRY-box containing protein 9a	*sox9a*	11.47	0.4	1.41	Testis
DN31258_c1_g1	SRY-box containing protein 9b	*sox9b*	5.47	0.26	0.83	Testis
DN36334_c0_g1	SRY-box containing protein 10	*sox10*	22.41	0.09	0	Testis
DN33725_c0_g1	Doublesex and mab-3 related transcription factor 1	*dmrt1*	59.86	1.59	0.03	Testis
DN24271_c0_g1	Doublesex and mab-3 related transcription factor 3	*dmrt3*	1.84	0.1	0.07	Testis
DN83821_c0_g1	Doublesex and mab-3 related transcription factor 4	*dmrt4*	1.28	0	0	Testis
DN36880_c0_g1	Piwi-like RNA-mediated gene silencing 1	*piwil1*	339.61	27.19	13.41	Testis
DN40102_c0_g1	Piwi-like RNA-mediated gene silencing 2	*piwil2*	74.81	3.75	7.35	Testis
DN44440_c1_g1	Synaptonemal complex protein 1	*sycp1*	181.95	0.78	0.03	Testis
DN40831_c0_g1	Synaptonemal complex protein 2	*sycp2*	188.91	1.57	0.02	Testis
DN36753_c1_g1	Synaptonemal complex protein 3	*sycp3*	1211.17	40.18	34.97	Testis
DN35615_c0_g1	Outer dense fiber protein 2	*odf2*	69.53	1.31	3.58	Testis
DN35823_c0_g1	Outer dense fiber protein 3	*odf3*	1002.84	7.26	0.19	Testis
DN42637_c3_g1	Forkhead transcription factor H1	*foxh1*	14.91	263.37	214.04	Ovotestis
DN30601_c0_g1	Doublesex and mab-3 related transcription factor 2	*dmrt2*	1.19	3.13	2.53	Ovotestis
DN65452_c0_g1	Doublesex and mab-3 related transcription factor 5	*dmrt5*	0.15	0.17	0.74	Ovary
DN22524_c0_g1	Forkhead transcription factor L2	*foxl2*	0.47	0.92	3.18	Ovary
DN30279_c0_g3	SRY-box containing protein 3	*sox3*	0.83	8.38	17.65	Ovary
DN41965_c1_g1	Factor in the germline alpha	*figla*	8.95	175.86	176.32	Ovary
DN40052_c0_g1	Zona pellucida glycoprotein 3	*zp3*	4.01	118.88	132.57	Ovary
DN9204_c0_g1	Nuclear receptor subfamily 0 group B member 1	*dax1*	0.5	1.27	2.48	Ovary

### Validation of Gene Expression by RT-qPCR

**FIGURE 6:**
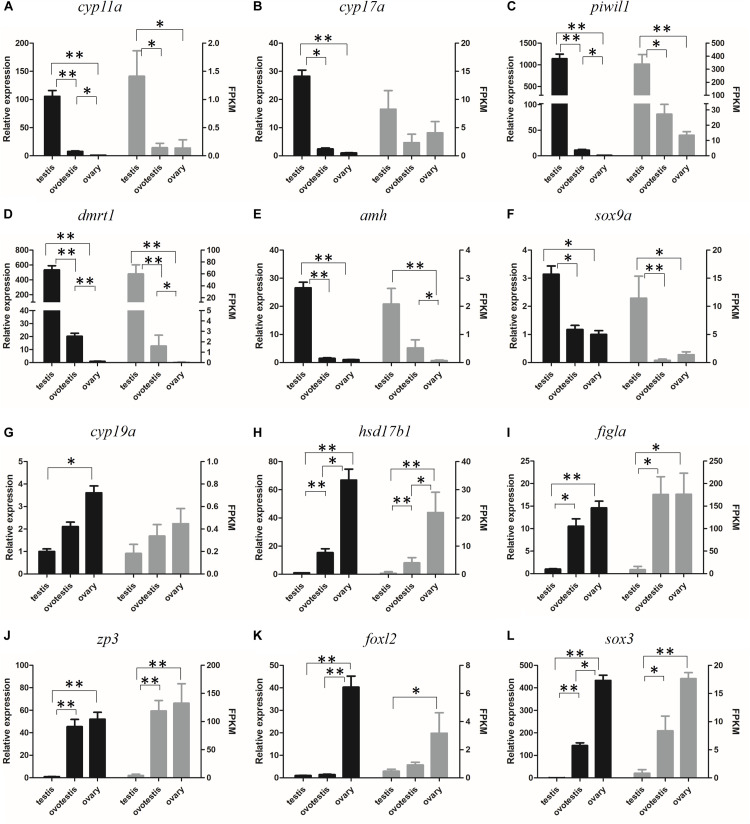
Verification of the expression patterns both in qRT-PCR (left) and RNA-seq (right). **(A)** *cyp11a*, **(B)** *cyp17a*, **(C)** *piwil1*, **(D)** *dmrt1*, **(E)** *amh*, **(F)** *sox9a*, **(G)** *cyp19a*, **(H)** *hsd17b1*, **(I)** *figla*, **(J)** *zp3*, **(K)** *foxl2*, **(L)** *sox3*. Student *t*-test, ^∗^*p* < 0.05 and ^∗∗^*P* < 0.01.

The reliability of the transcriptome data was validated by RT-qPCR analysis of 12 represented sex-related candidate genes. Consistent with our transcriptome data, qPCR analyses indicated that the expression of testicular development–related genes *cyp11a*, *cyp17a*, *dmrt1*, *amh*, *sox9a*, and *piwil1* were high in testis, gradually decreased in ovotestis, but almost undetectable in mature ovary. Whereas, the expression of ovarian development–related genes *cyp19a*, *hsd17b1*, *figla*, *zp3*, *foxl2*, and *sox3* were significantly upregulated in ovotestis and mature ovary in comparison with that in mature testis (Figure 6).

### *Dmrt1* and *Foxl2* Are Two Potential Sex Determining Genes in Yellowfin Seabream

**FIGURE 7:**
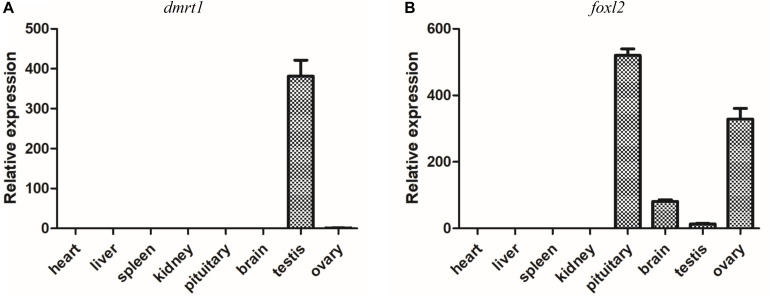
RT-qPCR detection of *dmrt1* **(A)** and *foxl2* **(B)** in different tissues. The relative expression is normalized by β-actin.

It has been demonstrated that *dmrt1* and *foxl2* are two important SD genes in vertebrates (Huang et al., 2017). Our transcriptome and RT-qPCR validation demonstrated the sexually dimorphic expression of *dmrt1* and *foxl2*, suggesting that they might be associated with sex differentiation in yellowfin seabream. Subsequently, the tissue distributions of *dmrt1* and *foxl2* transcripts were analyzed in various tissues, including heart, liver, spleen, kidney, pituitary, brain, testis, and ovary. As shown in Figure 7, *dmrt1* was exclusively expressed in the testis, indicating that it might play an important role in testis development in yellowfin seabream. The expression of *foxl2* was high in pituitary and ovary, moderate in the brain, but low in the testis, implying that *foxl2* might regulate ovary development through the brain-pituitary-ovary axis in yellowfin seabream.

## Discussion

In this study, we performed comprehensive transcriptomic analyses of the testis, ovotestis, and ovary in yellowfin seabream. By function analysis, we identified 55 key sex-related genes associated with steroidogenic hormones, wnt signaling pathway, germ cell development, and sex differentiation. Moreover, the testis-specific expression of *dmrt1* and brain-pituitary-ovary axis expression of *foxl2* indicates that they might play important roles in sexual differentiation in yellowfin seabream. Our results provide primary genetic information for gonadal development during sex reversal in yellowfin seabream. The identification of sex-related genes was the foundation for studying the sex differentiation and sex reversal in this species.

### Transcriptomic Overview of Different Gonad Developmental Stages in Yellowfin Seabream

Zhang et al. (2019) report *de novo* transcriptome data of the gonads in black seabream, a fish species that has a close phylogenetic relationship with yellowfin seabream. The N50 length (2194 bp) and average length (1062 bp) are similar in these two species, whereas the number of assembled unigenes (71,765) in yellowfin seabream is less than that (109,504) in black seabream, suggesting that their genetic information is different. Meanwhile, many unigenes were not annotated in the public databases, suggesting that there might be more novel transcripts in yellowfin seabream. Interestingly, two of the top 10 DEGs (*rsph* and *klhl10*) are found in both black seabream, and yellowfin seabream, suggesting they might play important roles in seabream sex reversal.

### The Expression Change of Sex-Related Signaling Pathway During Sex Reversal

Sex reversal is tightly regulated by steroid hormones, such as estrogen and testosterone (Gennotte et al., 2017; Otani et al., 2017; Han et al., 2018). Steroid hormones are synthesized through a series of isomerization and hydroxylation reaction catalyzed by many enzymes, such as cyp, and hydroxysteroid dehydrogenases families (Hilborn et al., 2017). It has been demonstrated that some CYP components play important roles in sexual differentiation (Uno et al., 2012). *Cyp11a* plays an important role in converting cholesterol to pregnenolone, which is the first step for the synthesis of steroid hormones. The sexual dimorphic expression of *cyp11a* has been characterized in several fish species (Nishiyama et al., 2015; Liang et al., 2018). *Cyp17* has both 17a-hydroxylase and 17, 20-lyase activities, which is required for producing androgens and estrogens. The sexual dimorphism of *cyp17a* has been observed in several fish species (Chen et al., 2010; Xiong et al., 2015). In zebrafish, *cyp17a1* mutants develop to all fertile males due to the decreased level of estrogen, demonstrating that *cyp17a1* is essential for estrogen production and ovarian maintenance (Zhai et al., 2018). *Cyp19a1a* plays a pivotal role in ovary development by converting the testosterone into estradiol (Guiguen et al., 2010; Fowler et al., 2011; Lambeth et al., 2013). Here, the downregulated expression of *cyp11a* and *cyp17a* and upregulated expression of *cyp19a1a* was found during testis-ovotestis-ovary sex reversal, indicating that they might be involved in sex differentiation in yellowfin seabream. *hsd17b1* and *hsd17b12* were essential for ovarian development by converting inactive estrone to active estradiol, whereas *hsd17b2* regulates testicular development by modification of testosterone (Hilborn et al., 2017, Hiltunen et al., 2019). Consistently, the testis-biased expression of *hsd17b2*, and ovary-biased expression of *hsd17b1* and *hsd17b12* were revealed in yellowfin seabream, indicating that they also might be involved in sexual differentiation as in the above studies.

Wnt4, one of the best characterized components in *wnt*/β-catenin signaling pathway, is required for initiating ovarian differentiation in mammal (Chassot et al., 2014; Roly et al., 2018). Recently, the critical role of wnt4 in folliculogenesis has been characterized in teleost, such as medaka (Li et al., 2012), spotted scat (Chen et al., 2016), orange-spotted grouper (Chen et al., 2015), and black seabream (Wu and Chang, 2009). During yellowfin seabream testis-to-ovary sex reversal, the expression of the wnt signaling pathway, including *wnt4*, *wnt5a*, *wnt9b*, *wnt16, axin2*, and *lef1*, were significantly upregulated, indicating that the wnt signaling played important roles in ovarian development in yellowfin seabream.

### Sex-Biased Expression of Germ Cell Markers in Yellowfin Seabream

The germ cell–specific *piwi* plays a crucial role in mammal spermatogenesis, male germ cell proliferation, differentiation, and meiosis control (Deng and Lin, 2002; Carmell et al., 2007; Houwing et al., 2007, 2008). Odfs are the components of sperm cytoskeletal structure, which are required for male fertility in mice and zebrafish (Hsu et al., 2010; Yang et al., 2014; Lehti and Sironen, 2017). *Zp3* encodes the zona pellucida glycoprotein, an important component that is required for oogenesis and fertilization in vertebrates (Mold et al., 2009; Sun et al., 2010; Chuang-Ju et al., 2011). It has been reported that *figla* plays an important role in folliculogenesis and ovarian differentiation (Nishimura et al., 2018; Qin et al., 2018). During yellowfin seabream sex reversal, the expression of *piwil1*, *piwil2*, *odf1*, and *odf2* were significantly decreased; meanwhile, the expression of *figla*, and *zp3* were dramatically increased, suggesting that they should contribute in sex differentiation in yellowfin seabream.

### Candidates of Sex Determination-Related and Sex Differentiation-Related Genes in Yellowfin Seabream

The DM-domain-containing genes might be the most important SD genes in animals (Matson and Zarkower, 2012). *dmrt1* plays a robust role in SD, male germ cell differentiation, and maintenance in mammal, bird, and fish species (Smith et al., 2009; Matson et al., 2011; Li et al., 2013; Lin et al., 2017; Zhang et al., 2016; Webster et al., 2017). *dmrt2* was highly expressed in male germ cells in Chinese tongue sole (Zhu et al., 2019); *dmrt2b* and *dmrt3* were dominantly expressed in the ovotestis of orange-spotted grouper (Lyu et al., 2019); *dmrt2*, *dmrt3*, *dmrt4*, and *dmrt5* were significantly upregulated during ovary-to-testis sex reversal in the swamp eel (Sheng et al., 2014). In yellowfin seabream, the testis-specific expression of *dmrt1*, *dmrt3*, and *dmrt4*; ovotestis-biased expression of *dmrt2*; and ovary-biased expression of *dmrt5* suggest that the *dmrt* family might play important roles in sex reversal (Table 4 and Figure 6D). Furthermore, the testis-specific tissue distribution of *dmrt1* implies its potential role in testis differentiation (Figure 7A).

The sox super family play multiple roles in many biological processes, including gonad development (Kamachi and Kondoh, 2013). The germ cell–specific *sox3* plays a significant role in gametogenesis, sex determination, and gonad differentiation in vertebrates (Takehana et al., 2014); *sox9* cooperates with *sox8* to protect the adult testis from male-to-female genetic reprogramming and complete degeneration (Vidal et al., 2001; Barrionuevo et al., 2016); *sox10* is an important factor in human disorders of sex development (Polanco et al., 2010); and *sox17* is critical for PGC formation (Irie et al., 2015). It has been implied that the expression of *sox3* was dynamic along with the process of sex reversal in protogynous hermaphroditic grouper (Yao et al., 2007) and protandrous hermaphroditic black seabream (Shin et al., 2009). *sox9a*, *sox9b*, and *sox10* were highly expressed in the testis, whereas *sox3* was mainly expressed in the ovary, suggesting that sox genes play complicated roles in sex differentiation in yellowfin seabream.

*foxl2*, the hallmark of granulosa cell, is one of earliest sexually dimorphic genes during ovarian development (Bertho et al., 2016; Okada et al., 2017), which plays a pivotal role in estradiol production through the endocrine system along brain-pituitary-gonad axis (Sridevi et al., 2012; Georges et al., 2014; Liu et al., 2019). Loss of *foxl2* results in dysregulation of estradiol and somatic cell reprogramming (Uhlenhaut et al., 2009) and even ovary-to-testis sex reversal (Li et al., 2013; Yang et al., 2017). Therefore, the brain-pituitary-ovary axis expression of *foxl2* indicated that *foxl2* might regulate ovarian differentiation in yellowfin seabream.

### The Mechanisms of Natural Sex Reversal in Fish Species

Natural sexual reversal is found in many fish species (Avise and Mank, 2009). This interesting phenomena and its potential underlying molecular mechanism have been characterized in some fish species. In orange-spotted grouper, the female-to-male sex reversal could be induced by overexpression of *amh*, which results in upregulation of testis development–associated genes, especially for those genes closely related with the synthesis of 11-ketotestosterone. Meanwhile, the decreased level of serum 17β-estradiol and the repression of ovarian development and maintenance-related genes were also found in *amh* overexpressed orange-spotted grouper (Han et al., 2018). In rice-field eel, there is a high expression level of *foxl2* and *cyp19a1a* in the ovary prior to sex reversal. However, at the beginning of the ovary-to-testis sex reversal, a sharp decrease of *foxl2* and *cyp19a1a* in the ovotestis (Liu et al., 2009; Hu et al., 2014). In black seabream, the sex switch is controlled by a series of male-related genes (*dmrt1, piwi1, piwi2, sox9, sox30*, and *amh*) and female-related genes (*jnk1, vasa, wnt4, figla*, and *foxl2*), most of which were involved in the signaling pathway of sex steroid hormones. The sex reversal is initiated by the decrease of male-related genes and the increase of female-related genes (Zhang et al., 2019). In yellowfin seabream, our results indicate that the sex reversal might be closely associated with 55 sex-related genes, most of which were involved in the signaling network of sex steroid hormones, such as estradiol and testosterone. Therefore, the sex reversal of yellowfin seabream should be controlled by the change of sex hormone and the decrease of androgens but elevated levels of estrogens. On the basis of the present work and previous studies in other fish species, we could conclude that the sex switch is determined and maintained by the balance of sex hormones. Once the original balance (estrogens in protogynous fish and androgens in protandrous fish) is broken out, the sex reversal might be induced by the occurrence of the upregulation of opposite hormones (androgens in protogynous fish and estrogens in protandrous fish).

## Conclusion

To our best knowledge, this is the first report of large-scale RNA sequencing of testis, ovotestis, and ovary in yellowfin seabream. Our present work provides an important genetic resource and a global view of DEGs and signaling pathway change during sex reversal of yellowfin seabream. A total of 55 sex differentiation–associated candidates has been obtained from the transcriptome data, including steroid biosynthesis–related genes (Cyp family and Hsd family), germ cell markers (*piwi*, *odf*, *figla*, and *zp3*), and the known SD factors, such as the DM domain containing family and Sox family. In addition, the testis-specific expression of *dmrt1* and the brain-pituitary-ovary axis expression of *foxl2* were characterized, suggesting that they might play important roles in sex differentiation in yellowfin seabream, and the specific function of these two genes deserve to be further investigated.

## Data Availability Statement

The datasets presented in this study can be found in online repositories. The names of the repository/repositories and accession number(s) can be found in the article/Supplementary Material.

## Ethics Statement

The animal study was reviewed and approved by Ethics Committee of Sun Yat-sen University. Written informed consent was obtained from the owners for the participation of their animals in this study.

## Author Contributions

JL and SL conceived and designed the experiments. SL and WF performed the experiments. GL, PH, and DG performed bioinformatics analysis. JH and JX contributed to discussion of the final results. SL wrote the manuscript. All authors contributed to the article and approved the submitted version.

## Conflict of Interest

The authors declare that the research was conducted in the absence of any commercial or financial relationships that could be construed as a potential conflict of interest.

## Footnotes

1 http://github.com/jstjohn/SeqPrep

2 http://github.com/najoshi/sickle

3 http://github.com/trinityrnaseq/trinityrnaseq

4 http://hibberdlab.com/transrate

5 http://weizhongli-lab.org/cd-hit/

6 http://busco.ezlab.org

7 http://deweylab.github.io/RSEM/

8 http://primer3.ut.ee
