# Supplementary material for: Gonadal Transcriptome Analysis of Sex-Related Genes in the Protandrous Yellowfin Seabream (Acanthopagrus latus)
Source: Front Genet. 2020 Jul 16;11:709. doi: 10.3389/fgene.2020.00709 (PMC7378800; doi:10.3389/fgene.2020.00709)
Supplement: Supplementary file 1 [file Table_1.DOCX]

**Table S1. The primer sequences used for RT-qPCR.**

| **Name** | **Sequence (5’ to 3’)** |
| --- | --- |
| Aldmrt1-F | GCCACAACATGTCCTCTCAGTA |
| Aldmrt1-R | CTCAGAGTTAACCAGGGAGCTG |
| Alfoxl2-F | GCCTCACTCTGTCCGGTATTTA |
| Alfoxl2-R | CCCCTTCTCAAACATGTCCTCA |
| Alsox3-F | ATGGAAACCGAGATCAAGACCC |
| Alsox3-R | CTCAGAGTTGTGCATTTTGGGG |
| Alamh-F | GTCCTACTCGAGCTCACTAACG |
| Alamh-R | CCATGTACTGTGTTTCCCCTGA |
| Alcyp19a-F | CTGGAGAGCTTCATCAACGAGT |
| Alcyp19a-R | GGATTTCATCATCACCATGGCG |
| Alsox9a-F | CAGAGAACCACCCAGATCAAGA |
| Alsox9a-R | CGTGGCTGTAGTAGGAGTTGG |
| Alpiwil1-F | AAAAGAGATGCGTGGGATGTCT |
| Alpiwil1-R | AGAGACTCCTGCTGATCCTCAT |
| Alfigla-F | ACTAATGCGACCAGACCGTAAG |
| Alfigla-R | CAAGTCATCCCCTCTCCATAGC |
| Alhsd17b1-F | TCACGGTCTCCCTTTTAACGAG |
| Alhsd17b1-R | CAGTGCTGCAGGTATCTCTCAT |
| Alcyp11a-F | AGATACAAAGAAGCGGCCAGAA |
| Alcyp11a-R | CCAAACTTTCGCTCCAATGTGT |
| Alcyp17a-F | AAGGCAGACTACAGTGACCATG |
| Alcyp17a-R | CACCTTACTGTCCAACTCCTCC |
| Alzp3-F | TTACTTCATGCAGAGGAGCCAG |
| Alzp3-R | CTCACAGCAGCTACACACCTTA |
| Alactin-F | ACACCTTCTACAACGAGCTGAG |
| Alactin-R | GTCTCGAACATGATCTGGGTCA |
