# Supplementary material for: Gonadal Transcriptome Analysis of Sex-Related Genes in the Protandrous Yellowfin Seabream (Acanthopagrus latus)
Source: Front Genet. 2020 Jul 16;11:709. doi: 10.3389/fgene.2020.00709 (PMC7378800; doi:10.3389/fgene.2020.00709)
Supplement: Supplementary file 2 [file Table_2.DOCX]

**Table S2. The overview of transcriptome data of yellowfin seabream.**

| **Sample** | **Raw reads** | **Raw bases** | **Clean reads** | **Clean bases** | **Error rate (%)** | **Q20 (%)** | **GC content (%)** |
| --- | --- | --- | --- | --- | --- | --- | --- |
| T_1 | 66574928 | 10.0G | 66223282 | 9.8G | 0.0239 | 98.51 | 48.84 |
| T_2 | 68804116 | 10.4G | 68387226 | 10.0G | 0.0231 | 98.82 | 50.26 |
| T_3 | 65509486 | 9.9G | 65085252 | 9.7G | 0.0234 | 98.72 | 50.66 |
| OT_1 | 61581128 | 9.2G | 60826162 | 8.9G | 0.0253 | 97.9 | 52.02 |
| OT_2 | 52166556 | 7.8G | 51734870 | 7.6G | 0.0167 | 97.93 | 51.94 |
| OT_3 | 45537844 | 6.8G | 45252758 | 6.7G | 0.0164 | 98.17 | 51.81 |
| O_1 | 43445888 | 6.5G | 43117998 | 6.4G | 0.0175 | 97.62 | 48.36 |
| O_2 | 59439728 | 9.0G | 58917948 | 8.8G | 0.0239 | 98.48 | 51.93 |
| O_3 | 66190392 | 10.0G | 65665300 | 9.8G | 0.0235 | 98.64 | 51.67 |
