# Supplementary material for: Gonadal Transcriptome Analysis of Sex-Related Genes in the Protandrous Yellowfin Seabream (Acanthopagrus latus)
Source: Front Genet. 2020 Jul 16;11:709. doi: 10.3389/fgene.2020.00709 (PMC7378800; doi:10.3389/fgene.2020.00709)
Supplement: Supplementary file 3 [file Table_3.DOCX]

**Table S3. Statistics of transcriptome assembly**

| **Type** | **Resource** |
| --- | --- |
| Total transcripts, n | 98732 |
| Total unigenes, n | 71765 |
| Total sequence base, bp | 118536405 |
| Largest length, bp | 17019 |
| Smallest length, bp | 201 |
| Average length, bp | 1201 |
| N50 length, bp | 2032 |
| E90N50 length, bp | 2707 |
| GC percent, % | 47.17 |
