# Supplementary material for: Gonadal Transcriptome Analysis of Sex-Related Genes in the Protandrous Yellowfin Seabream (Acanthopagrus latus)
Source: Front Genet. 2020 Jul 16;11:709. doi: 10.3389/fgene.2020.00709 (PMC7378800; doi:10.3389/fgene.2020.00709)
Supplement: Supplementary file 4 [file Table_4.DOCX]

**Table S4. Summary statistics of annotation for unigenes in public databases.**

| **Type** | **Number of unigenes** | **Percentage (%)** |
| --- | --- | --- |
| Annotated in NR | 29252 | 40.76 |
| Annotated in Swiss-Prot | 25425 | 35.43 |
| Annotated in Pfam | 22732 | 31.68 |
| Annotated in COG | 13183 | 18.37 |
| Annotated in GO | 9522 | 13.27 |
| Annotated in KEGG | 11672 | 16.26 |
| Annotated in all Databases | 3480 | 4.85 |
| Annotated in at least one Database | 30587 | 42.62 |
| Total unigenes | 71765 | 100 |
