# Supplementary material for: Gonadal Transcriptome Analysis of Sex-Related Genes in the Protandrous Yellowfin Seabream (Acanthopagrus latus)
Source: Front Genet. 2020 Jul 16;11:709. doi: 10.3389/fgene.2020.00709 (PMC7378800; doi:10.3389/fgene.2020.00709)
Supplement: Supplementary file 5 [file Table_5.DOCX]

**Table S5. Top 10 up-regulated and down-regulated genes in the ovary compared with the testis.**

| **Sequence ID** | **Annotation** | **Gene name** | **Log2FC**  **(ovary/ testis)** |
| --- | --- | --- | --- |
| DN42580_c2_g1 | Sodium/glucose cotransporter 4-like | *Sglt4l* | 10.97 |
| DN27243_c0_g1 | synaptic vesicle 2-related protein-like | *Svop2l* | 10.16 |
| DN23463_c0_g1 | probable pancreatic secretory proteinase inhibitor isoform X2 | *Pspi* | 10 |
| DN35542_c0_g1 | toll-like receptor 2 type 2 | *Tlr2* | 9.85 |
| DN35544_c0_g1 | fatty acid-binding protein, adipocyte-like | *Fabp* | 9.51 |
| DN44467_c0_g2 | Tubulin alpha-1C chain-like | *Tuba1c* | 9.08 |
| DN22784_c0_g1 | diencephalon/mesencephalon homeobox protein 1 | *Dmbx1* | 8.75 |
| DN38926_c0_g2 | Solute carrier family 22 member 13 | *Slc22a13* | 8.74 |
| DN41124_c2_g1 | Leukemia NUP98 fusion partner 1 isoform X1 | *Lnp1* | 8.7 |
| DN11175_c0_g1 | nicotinamide/nicotinic acid mononucleotide adenylytransferase 2 isoform X1 | *Nmat2* | 8.43 |
| **Sequence ID** | **Annotation** | **Gene name** | **Log2FC**  **(ovary/ testis)** |
| DN32964_c0_g1 | protein FAM166B-like | *Fam166bl* | -14.77 |
| DN39140_c0_g1 | coiled-coil domain-containing protein 42 like-2-like | *Ccdc42l* | -14.63 |
| DN27344_c0_g1 | IQ domain-containing protein G | *Iqcg* | -14.44 |
| DN42167_c0_g1 | radial spoke head 10 homolog B2 isoform X2 | *Rsph10b2* | -14.01 |
| DN42438_c0_g1 | mitochondria-eating protein | — | -13.83 |
| DN44044_c1_g2 | IQ motif-containing protein H | *Iqch* | -13.81 |
| DN38557_c1_g1 | cingulin-like | *Cgnl* | -13.75 |
| DN43056_c0_g2 | Ankyrin repeat and MYND domain- containing protein 1 | *Ankmy1* | -13.71 |
| DN40218_c0_g1 | radial spoke head protein 6 homolog A-like | *Rsph6a* | -13.68 |
| DN17044_c0_g1 | testis-expressed protein 49-like | *Tex49l* | -13.64 |
