# Supplementary material for: Gonadal Transcriptome Analysis of Sex-Related Genes in the Protandrous Yellowfin Seabream (Acanthopagrus latus)
Source: Front Genet. 2020 Jul 16;11:709. doi: 10.3389/fgene.2020.00709 (PMC7378800; doi:10.3389/fgene.2020.00709)
Supplement: Supplementary file 6 [file Table_6.DOCX]

**Table S6. Top 10 up-regulated and down-regulated genes in the ovary compared with the ovotestis.**

| **Sequence ID** | **Annotation** | **Gene name** | **Log2FC**  **(ovary/ ovotestis)** |
| --- | --- | --- | --- |
| DN19842_c0_g1 | opsin-5-like | *Opn5l* | 9.26 |
| DN42380_c1_g1 | Guanosine monophosphate reductase 1 | *Gmpr1* | 8.08 |
| DN42295_c0_g1 | C-type lectin domain family 6 member A-like | *Clec6al* | 7.64 |
| DN17148_c0_g1 | interleukin-12 receptor subunit beta-2-like | *Il12rb2l* | 7.31 |
| DN26965_c0_g1 | noggin-3-like | *Nog3l* | 7.25 |
| DN18967_c0_g1 | serine/threonine-protein kinase H1-like | *Stkh1l* | 7.11 |
| DN23250_c0_g1 | cholesterol 25-hydroxylase-like protein 1, member 1 | *Ch25h* | 7.02 |
| DN29165_c0_g1 | Nuclear factor kappa-B inhibitor delta | *Nfkbid* | 6.61 |
| DN42580_c2_g1 | Sodium/glucose cotransporter 4-like | *Sglt4l* | 6.51 |
| DN30695_c0_g1 | complement component C9 | *C9* | 6.45 |
| **Sequence ID** | **Annotation** | **Gene name** | **Log2FC**  **(ovary/ ovotestis)** |
| DN29644_c0_g1 | serine/threonine-protein kinase/endoribonuclease IRE1-like | *Ire1l* | -10.62 |
| DN26381_c0_g1 | Complement C1q-like protein 4 | *C1ql4* | -9.31 |
| DN27875_c0_g2 | Homeobox protein MOX-2 | *Mox2* | -9.07 |
| DN2133_c0_g1 | sodium/hydrogen exchanger 2-like | *Slc9a2l* | -9.06 |
| DN1528_c0_g1 | basement membrane-specific heparin sulfate proteoglycan core protein-like | *Hspgl* | -8.97 |
| DN40636_c2_g2 | allantoinase, mitochondrial-like | *Aln* | -8.93 |
| DN32964_c0_g1 | protein FAM166B-like | *Fam166bl* | -8.82 |
| DN35170_c0_g1 | angiogenin | *Ang* | -8.79 |
| DN35050_c0_g1 | trans-L-3-hydroxyproline dehydratase isoform X1 | *L3hypdh* | -8.68 |
| DN15114_c0_g1 | Elongation factor 1-alpha 1 | *Eef1a1* | -8.61 |
